# Identification of the *ACTB* p.Ser348Leu *de novo* variant in individuals with syndromic neonatal diabetes

**DOI:** 10.1016/j.ebiom.2026.106286

**Published:** 2026-05-09

**Authors:** Suhel Ahmed, Victoria Lewis, James Russ-Silsby, Matthew N. Wakeling, Maria Thunander, Maritza Vivanco, Richard Caswell, Andrew T. Hattersley, Pamela Bowman, Kashyap Patel, Sarah E. Flanagan, Elisa De Franco

**Affiliations:** a Department of Clinical and Biomedical Sciences, Faculty of Health and Life Sciences, University of Exeter, UK; b Department of Clinical Sciences, Endocrinology and Diabetes, Lund University, Lund, Sweden; c Department of Research and Development, Region Kronoberg, Sweden; d Hospital de Niños Dr. Roberto del Río, University of Chile, Santiago, Chile; e Exeter Genomics Laboratory, Royal Devon University Healthcare NHS Foundation Trust, Exeter, UK

**Keywords:** Neonatal diabetes, ACTB, Beta-actin, Genome sequencing

## Abstract

**Background:**

Identifying novel genetic causes of diabetes in the first 6 months of life (neonatal diabetes) can highlight genes and pathways essential for pancreatic beta-cell development and function in humans. Our aim was to uncover genetic aetiologies of neonatal diabetes.

**Methods:**

We performed genome sequencing in 38 probands diagnosed with neonatal diabetes without an identified genetic cause, and their unaffected parents. Genes with *de novo* coding variants in ≥2 probands were followed up. Replication was performed in a separate cohort of 288 genetically unresolved individuals diagnosed with neonatal diabetes.

**Findings:**

The *de novo ACTB* (p.Ser348Leu) variant was identified in two unrelated individuals. Both had diabetes onset soon after birth (1 and 8 days), low birthweight (−3.22SD and −3.98SD), and extra-pancreatic features (hearing loss and developmental delay in one; intestinal atresia in the other). The same *ACTB* (p.Ser348Leu) variant was reported in seven individuals in the literature; one individual had confirmed neonatal diabetes and a further two had hyperglycaemia. Extra-pancreatic features were similar to our probands (hearing loss in 3/7; neurodevelopmental features in 4/7; gastrointestinal atresia in 6/7). In total, 5/9 individuals with the *ACTB* (p.Ser348Leu) variant had neonatal diabetes or hyperglycaemia. None of the 96 cases with other *ACTB* pathogenic variants in the Human Gene Mutation Database had diabetes.

**Interpretation:**

The identification of 3 individuals (two in this report and one from the literature) with neonatal diabetes and a *de novo ACTB* p.(Ser348Leu) variant supports *ACTB* as a previously unrecognised neonatal diabetes aetiological gene, most likely through a variant-specific mechanism.

**Funding:**

Diabetes UK; EFSD/NNF; NIHR, Wellcome Trust.

**Research in context**

**Evidence before this study**

Neonatal diabetes is a rare monogenic condition typically diagnosed before six months of age. A recent review reported more than 40 genetic causes of neonatal diabetes. The *ACTB* gene was not listed as a neonatal diabetes causative gene in this review. In the OMIM (Online Mendelian Inheritance in Man) database autosomal dominant *ACTB* variants have been reported to cause a spectrum of genetic conditions, including Baraitser-Winter syndrome (MIM #243310), Deafness-Dystonia syndrome (MIM #607371) and Thrombocytopenia with or without dysmorphic features (MIM #620475). Diabetes has not so far been associated with any ACTB-related disorders.

**Added value of this study**

We report the identification of a *de novo* heterozygous c.1043C>T p.(Ser348Leu) variant in the *ACTB* gene in two unrelated individuals diagnosed with neonatal diabetes and additional extra-pancreatic features.

A review of the clinical features of 103 individuals with *ACTB* variants listed in the Human Gene Mutation Database identified three individuals, all heterozygous for the same *ACTB* p.(Ser348Leu) variant, who were also diagnosed with neonatal diabetes or hyperglycaemia. Our findings reveal a significant enrichment of neonatal diabetes in individuals heterozygous for the *ACTB* p.(Ser348Leu) variant and expands the spectrum of clinical features associated with *ACTB*-related disorders.

**Implications of all the available evidence**

Our results support a role of the *ACTB* p.(Ser348Leu) variant in the aetiology of neonatal diabetes, and we therefore recommend the *ACTB* gene is added to routine genetic testing for neonatal diabetes.

## Introduction

Neonatal diabetes mellitus (NDM) is a clinically diverse monogenic condition with an incidence of ∼1 in 100,000 live births, typically diagnosed within the first six months of life.^1^^,^^2^ Depending on diabetes progression, NDM can be classified into two categories: permanent neonatal diabetes, where the condition persists throughout life, or transient neonatal diabetes, where diabetes initially resolves shortly after diagnosis but typically recurs later in life. A third subtype, syndromic NDM, is characterised by the presence of extra-pancreatic features.^3^

NDM is a genetically heterogenous disorder, with over 40 known genetic causes, with 12 novel aetiologies identified between 2018 and 2024.^4^ Much of this success can be attributed to the application of next generation sequencing approaches such as exome and genome sequencing, which have enabled the transition from a candidate-gene centric approach to a gene-agnostic method. One of the most effective approaches has been the use of proband-parents trio analysis, where an affected proband is sequenced alongside their unaffected, unrelated parents to identify *de novo* variants. This strategy has resulted in the identification of at least 5 novel dominant causes of NDM.^5^, ^6^, ^7^, ^8^, ^9^

The heterogeneity of genetic causes of NDM reflects the broad range of biological mechanisms by which diabetes can arise, including impaired pancreatic beta-cell function, beta-cell loss due to dysregulated endoplasmic reticulum stress or autoimmune destruction, and disruption of pancreatic development leading to a reduced number or absence of beta-cells.^10^ The genetic heterogeneity of NDM is further broadened by the range of underlying molecular mechanisms. Loss-of-function, via biallelic inactivation (e.g., *EIF2AK3*^11^) or haploinsufficiency (e.g., *GATA6*^7^), resulting in a deficiency of the protein product is the genetic mechanism underlying the majority of genetic causes. Gain-of-function (e.g., *ABCC8*^12^, *KCNJ11*^13^) and dominant-negative variants (e.g., *INS*^14^) which alter or disrupt wild-type protein function, are also common genetic mechanisms observed in NDM.

Variant-specific mechanisms are increasingly recognised in NDM, where only certain genetic variants lead to the disorder, while other variants in the same gene give rise to a clinically distinct condition. For instance, the dominant *CNOT1* p.(Arg535Cys) variant causes NDM and exocrine pancreatic insufficiency,^5^ whereas other heterozygous loss-of-function *CNOT1* variants result in Vissers-Bodmer syndrome, a neurodevelopmental disorder not involving diabetes.^15^ Similarly, specific homozygous missense variants in the *TARS2*^16^ and *FICD*^17^ genes have been identified as causes of syndromic NDM through variant-specific mechanisms, whereas other recessive variants in the same genes lead to syndromic conditions that do not include diabetes.

Uncovering the genes and genetic mechanisms underlying NDM can provide insights into human pancreatic development and beta-cell biology. In this study, we performed trio-based genome sequencing in individuals with NDM without a prior genetic diagnosis and identified a specific *de novo ACTB* missense variant in two unrelated individuals with NDM.

## Methods

### Patient cohort

We studied 38 individuals diagnosed with NDM, defined as treatment-requiring hyperglycaemia with onset before six months of age, in whom all known genetic causes had been excluded. These individuals were recruited internationally and referred to the Exeter genomics laboratory (United Kingdom) where genetic testing and genetic analysis were performed. Genetic testing used a targeted next generation sequencing panel of all known NDM causative genes, along with methylation-specific MLPA for abnormalities at the 6q24 locus. Methodological details have been published previously.^6^

The median age at diagnosis of diabetes for the 38 individuals was 3 weeks. All were born to unrelated, unaffected parents. Extra-pancreatic features, consistent with syndromic NDM, were reported in 63% (24/38) of cases.

### Gene discovery analysis

Genome sequencing was performed on 38 probands and their unaffected parents. DNA was extracted from peripheral blood leukocytes collected in EDTA-blood tubes using a chemagic STAR system (Hamilton company) with the chemagic STAR DNA blood extraction kit (CMG-1796 (Revvity)). Sequencing was performed either by Illumina on an Illumina HiSeq 2500 (n = 2 trios) or by BGI with BGISEQ-500 (n = 36 trios) with a mean read depth of 37.03 (standard deviation 5.54) across the 114 samples. Sequencing libraries were prepared according to platform requirements. Sequencing data was aligned to the GRCh37/hg19 human reference genome using Burrows-Wheeler Aligner-Maximal Exact Matches. Data processing followed the Genome Analysis Toolkit (GATK; RRID:SCR_001876) best practices.^18^ Variants were identified using GATK HaplotypeCaller and annotated using Alamut Batch version 1.11 (Sophia Genetics). Quality control filters were applied to exclude variants with a QualByDepth (QD) score <2 or those supported by fewer than 5 reads.

Given that all probands were born to unrelated, unaffected parents we prioritised *de novo* heterozygous coding variants for follow-up, focussing on genes with such variants identified in at least two unrelated probands. Only *de novo* variants with an allele balance of >0.25 that were absent from gnomAD v4.1.0^19^ were considered for further analysis.

Follow-up of candidate variants was conducted by screening a replication cohort of 288 individuals with NDM not included in the initial discovery analysis and in whom known genetic causes of NDM had also been excluded. Variant screening was conducted using a targeted next generation sequencing assay incorporating RNA baits for the genes of interest (n = 71), exome sequencing (n = 41) or genome sequencing (n = 176). For samples analysed by exome sequencing, enrichment of exonic sequences and known non-coding regions linked to neonatal diabetes was performed using a custom TWIST Bioscience Exome kit (version 2) (RRID:SCR_025817). Following library preparation, sequencing was conducted using the Illumina (RRID:SCR_010233) NovaSeq platform. The resulting sequence reads were aligned to the GRCh37/hg19 human reference genome using the Burrows-Wheeler Aligner (BWA). Following alignment, variants were identified using the GATK (RRID:SCR_001876) best practice guidelines^18^ and annotated using Ensembl Variant Effect Predictor (RRID:SCR_007931). For targeted next generation sequencing, in solution targeted enrichment was performed using either a custom Agilent SureSelect or TWIST Bioscience (RRID:SCR_025817) exon-capture assay with RNA baits for all known neonatal diabetes genes and candidate genes. Sequencing following library preparation was performed on either the Illumina (RRID:SCR_010233) NextSeq 500 or NextSeq 550 platforms. Sequencing data was processed as described for the exome sequencing analysis. Further details on this assay have been published before.^20^ SavvyCNV was used to detect copy number variation in next generation sequencing data.^21^ All three sequencing methods provided sufficient coverage (>20×) to identify variants in candidate genes.

Prioritised variants were classified using the American College of Medical Genetics guidelines (ACMG).^22^ The *in-silico* tools REVEL and AlphaMissense were used to assess the predicted effect of missense variants.^23^^,^^24^

### Follow-up of the *ACTB* p.(Ser348Leu) variant

We searched PubMed and Google Scholar to identify any previous reports of the *ACTB* p.(Ser348Leu) variant in the literature. In publications where an individual was identified with the variant, the clinical data available was analysed for any mention of diabetes or hyperglycaemia. If either term was mentioned, we next investigated if this was detected in the neonatal period. Any additional details, such as treatment type and dose, that could be obtained are shown in Table 1. Individuals with neonatal hyperglycaemia with confirmed treatment requirement were classified as having NDM for subsequent analysis (n = 1).

**Table 1:** Clinical details of the nine individuals heterozygous for the *ACTB* p.(Ser348Leu) variant.

Reference	Exeter P1	Exeter P2	Sibbin et al.	Sibbin et al.	Jarvela et al.	Brea-Fernandez et al.	Fakhro et al.	Hill et al.	Tsujimoto et al.
Birthweight (g)/Gestation (weeks)	1370/37	1598/37	2375/29.5	1210/29.5	2225/36	NR/NR	NR/NR	NR/32	NR/NR
Neonatal diabetes or Hyperglycaemia	Neonatal diabetes	Neonatal diabetes	NR	Hyperglycaemia	NR	NR	Hyperglycaemia	Neonatal diabetes	NR
Age at diabetes or hyperglycaemia report	6 h	8 days	NA	<23 days	NA	NA	NR	1 day	NA
Age at last assessment	1 month	24.9 years	1 day	23 days	14 years	NR	NR	6 weeks	9 years
Insulin dose (U/kg/day)	0.02	0.51	NA	NR	NA	NA	NR	Subcutaneous insulin; dose NR	NA
Hearing loss	NR	Yes	NR	NR	Yes	Yes	NR	NR	Yes
Gastrointestinal atresia/stenosis	Intestinal atresia	NR	Small bowel atresia	Jejunal atresia (apple-peel deformity)	Duodenal atresia (apple-peel deformity)	NR	Jejunal atresia (apple-peel deformity)	Jejunal atresia	Stenosis
Neurodevelopmental features	NR	Intellectual disability	NR	NR	Intellectual disability, seizures	Intellectual disability	NR	Hypotonia	Intellectual disability
Other features	Renal dysplasia, choledochal cyst, aortic coarctation. Deceased.	Hearing aids required, scoliosis (underwent surgery).	Generalised subcutaneous oedema, craniofacial features, pulmonary hypoplasia, single coronary ostium. Deceased.	Craniofacial features, congenital lung malformations, dilated stomach, megakaryocytes. Deceased	Craniofacial features, coloboma, pachygyria, bicornuate uterus, motor clumsiness.	NR	NR	Respiratory distress, craniofacial features, hypoplastic nipples, lack of subcutaneous fat, oedema, leukopenia, anaemia, low pancreatic elastase, renal pelviectasis, unilateral dilated ureter.	Craniofacial features, ptosis, otitis media, hypertelorism, ventricular septal defect, atrial septal defect, cryptorchidism.

The two individuals identified in this study are identified as Exeter P1 and P2. NA = Not applicable, NR = Not reported.

### Phenotype enrichment analysis

To assess whether NDM had been reported as a feature in other individuals with *ACTB*-related disease, all *ACTB* disease-causing variants listed in the Human Gene Mutation Database (HGMD) were retrieved along with their associated publications (date accessed 26/10/2023). A total of 41 publications containing individual-level clinical data were selected for review. Each publication was evaluated for reports of hyperglycaemia or diabetes in the neonatal period consistent with a diagnosis of NDM in patients carrying a disease-causing *ACTB* variant. The phenotype enrichment analysis included all individuals from: selected publications on other *ACTB* disease-causing variants (n = 96), publications previously reporting the *ACTB* p.(Ser348Leu) variant (n = 7), and the individuals with the *ACTB* p.(Ser348Leu) variant identified by our study (n = 2).

### In silico protein–protein interaction and protein modelling analysis

The PDBe-KB database^25^ was inspected to identify protein–protein interactions of Ser348 or its neighbours, Ala347 or Leu349; the p.(Ser348Leu) variant was then analysed using FoldX v5.0^26^ (RRID:SCR_008522) to establish the impact of the variant on the underlying stability of ACTB and the change in interaction energy between ACTB and its binding partner.

### Statistical analysis

Inclusion into the genome-sequencing discovery cohort (n = 38) was based on a) a diagnosis of diabetes before 6 months of age without a known genetic cause and b) parents being unrelated and unaffected. Inclusion in the follow up cohort was based on a diagnosis of diabetes before 6 months of age without a known genetic cause. As we are investigating individuals with rare monogenic disease, sample size calculation was not applicable.

A Fisher’s exact test was used to determine whether NDM, hyperglycaemia, gastrointestinal atresia/stenosis, thrombocytopenia and neurodevelopmental features were significantly over-represented in individuals with the *ACTB* p.(Ser348Leu) variant (n = 9) compared to individuals with other disease-causing *ACTB* variants listed in HGMD (n = 96; only individuals with sufficient clinical data available were considered). The Bonferroni correction was applied to account for testing of multiple clinical features and adjusted p-values reported. The Bonferroni adjusted significance threshold was set to 0.01.

### Ethics

Informed consent for genetic research was obtained from all participants and/or their parents or guardians at referral to the Exeter Genomics Laboratory. The study complied with the principles of the Declaration of Helsinki. The study received ethical approval from the Genetic Βeta-cell Research Bank, Exeter, U.K, which itself received ethical approval from the North Wales Research Ethics Committee (REC reference: 517/WA/0327).

### Role of funders

The funders had no role in the study design, data collection, analyses, interpretation, or the writing of the manuscript.

## Results

### Identification of a shared *de novoACTB* missense variant in 2 individuals with NDM

A total of 30 *de novo* variants were identified in the 38 probands (average 0.79 per trio, range 0–3). Only one gene, *ACTB* encoding beta actin, was found to harbour *de novo* variants in at least 2 individuals.

**Fig. 1:**
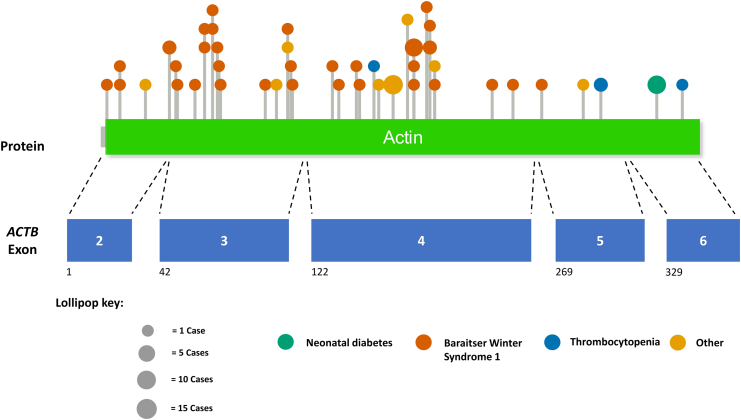
**Lollipop plot of pathogenic missense variants located across the *ACTB* gene as reported in HGMD.** The location of the heterozygous p.(Ser348Leu) variant identified in our patients is indicated by the green colour. Lollipops are colour-coded by the phenotype associated with each variant in HGMD. Missense variants and their associated phenotypes are provided in Supplementary Table S1. The size of the lollipop corresponds to the number of cases reported for each variant in HGMD. Numbers below the exons indicate amino acid position corresponding to exon start. HGMD = Human gene mutation database.

A *de novo* heterozygous c.1043C>T p.(Ser348Leu) variant in exon 6 of the *ACTB* gene was detected in two unrelated individuals (Fig. 1). The p.(Ser348Leu) variant is predicted to be deleterious by REVEL (score = 0.9, strong likelihood that the variant is deleterious) and AlphaMissense (pathogenicity score >0.99). The variant is absent in 807,162 individuals in gnomAD v4.1.0.^19^ Sequence analysis of the *ACTB* gene in the replication cohort of 288 probands with NDM of unknown genetic cause did not identify any further individuals with heterozygous, rare *ACTB* variants.

In HGMD, 94 variants (including single nucleotide variants, indels, and large deletions) affecting *ACTB* have been reported as disease-causing, of which 54 are missense variants (Fig. 1). Dominant missense *ACTB* variants causing gain-of-function have been reported to cause Baraitser-Winter syndrome 1 (BWS1) (MIM #243310) and Dystonia-deafness syndrome 1 (MIM #607371). In contrast, heterozygous loss-of-function *ACTB* variants cause a pleiotropic developmental disorder with characteristic dysmorphic features,^27^ while loss-of-function variants specifically in exons 5 and 6 give rise to a distinct condition characterised by thrombocytopenia, dysmorphic features and developmental delay (MIM #620475).

The heterozygous p.(Ser348Leu) variant has been previously reported as causing BWS1 in seven cases in the literature,^28^, ^29^, ^30^, ^31^, ^32^, ^33^ and it is classified as pathogenic/likely pathogenic in ClinVar (RCV000370520.30, 9 submissions). Using all available evidence and following the ACMG variant classification guidelines, we classified the *ACTB* p.(Ser348Leu) variant as pathogenic in both probands.

### Clinical features of individuals heterozygous for the *de novoACTB* p.(Ser348Leu) variant

Clinical details for the two probands identified in this study and previously reported individuals heterozygous for the *ACTB* p.(Ser348Leu) variant are provided in Table 1.

Patient 1 was a female proband who presented with severely reduced birthweight (1370 g at 37 weeks gestation; −3.98 SD) and a diagnosis of diabetes 6 h after birth. She had multiple extra-pancreatic features including intestinal atresia and renal dysplasia. She was treated with insulin (0.02 U/kg/day) until her death at 1 month of age following surgery for a choledochal cyst.

Patient 2, a female proband, had similarly reduced birthweight (1598 g at 37 weeks gestation; −3.22 SD) and was diagnosed with diabetes at 8 days. She was treated with insulin since diagnosis and her insulin dose at referral (at 6 years and 4 months old) was 0.53 U/kg/day. Her insulin dose at last assessment (aged 25) was 0.51 U/Kg/day with excellent glycaemic control (HbA1c 6.5%, 48 mmol/mol). Her exocrine pancreatic function, assessed when she was 12 years old, was normal (fecal elastase >200ug/g). She also has moderate intellectual disability, bilateral hearing loss and scoliosis which required surgery.

We next investigated the phenotype described in the seven individuals heterozygous for the *ACTB* p.(Ser348Leu) variant reported in the literature (individuals reported in ClinVar were not investigated due to clinical data being unavailable). Neonatal diabetes was diagnosed in one individual who was treated with subcutaneous insulin reported by Hill et al.^32^ Hyperglycaemia was reported in two additional individuals reported by Sibbin et al.^28^ and Fakhro et al.^31^ respectively. Hyperglycaemia was reported within the neonatal period in the individual reported by Sibbin et al. (<23 days, treatment information not available) whilst it is unclear whether this is the case in the individual reported by Fakhro et al. due to unavailability of individual data.^28^^,^^31^

In total, including the two patients identified in this study, 5/9 individuals with the *ACTB* p.(Ser348Leu) variant were diagnosed with hyperglycaemia, and 3/9 had a confirmed diagnosis of NDM (median age at diagnosis: 2 days, range: 6 h - 8 days, n = 3). Extra-pancreatic features were common in patients with the *ACTB* p.(Ser348Leu) variant, with gastrointestinal atresia identified in 7 (78%) and neurodevelopmental features in 5 (56%) individuals. In addition, one individual (reported by Hill et al.^32^) was reported to have pancreatic exocrine insufficiency (Table 1).

### Enrichment of NDM among patients with the *ACTB* p.(Ser348Leu) variant

Excluding the *ACTB* p.(Ser348Leu) variant, 68 disease-causing variants in 96 individuals, from 41 publications listed in HGMD, were investigated. None of these 96 individuals were reported to have hyperglycaemia or NDM. We tested if NDM and hyperglycaemia were observed significantly more frequently in individuals with the *ACTB* p.(Ser348Leu) variant. We found a statistically significant enrichment of hyperglycaemia and NDM in individuals harbouring the *ACTB* p.(Ser348Leu) variant when compared to all other disease-causing variants in the *ACTB* gene (5/9 vs 0/96, *Padj* = 6.52 × 10^−6^ and 3/9 vs 0/96 *Padj* = 0.002, respectively; Fisher’s exact test).

**Fig. 2:**
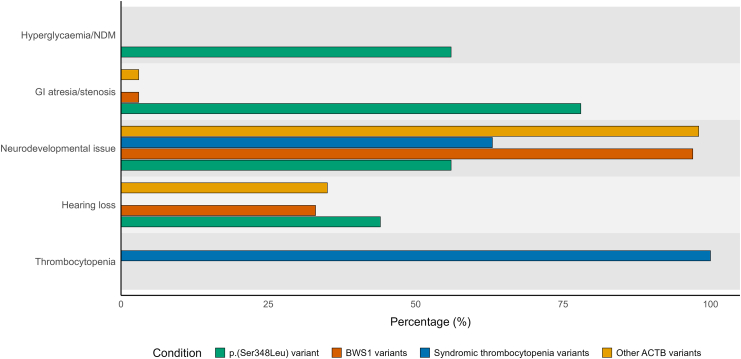
**A comparison of clinical features observed in individuals with the p.(Ser348Leu) *ACTB* variant vs other pathogenic *ACTB* variants.** Clinical features are grouped by the phenotypes described in Fig. 1: p.Ser348Leu variant (N = 9), BWS1 variants (N = 30), syndromic thrombocytopenia variants (N = 8), other *ACTB* variants (N = 58). GI = Gastrointestinal; NDM = Neonatal diabetes.

In addition to the enrichment for NDM, gastrointestinal atresia/intestinal stenosis is common amongst those with the p.(Ser348Leu) variant but rare in individuals with other pathogenic *ACTB* variants (7/9 vs 3/96, OR = 91.6, *Padj* = 8.99 × 10^−7^; Fisher’s exact test). Thrombocytopenia, typically associated with variants in exon 5 and 6 of *ACTB* was not observed in any individuals with the p.(Ser348Leu) variant, despite its location in exon 6. Neurodevelopmental features, the most common clinical manifestation of pathogenic *ACTB* variants, were present in individuals with the p.(Ser348Leu) variant at a lower frequency although this difference was not significant (5/9 vs 91/96, OR = 0.07, *Padj* = 0.015; Fisher’s exact test) (Fig. 2).

### In silico protein modelling of the *ACTB* p.(Ser348Leu) variant

To explore the potential effect of the *ACTB* p.(Ser348Leu) variant, in silico protein modelling was performed on interactions where Ser348 or the single residues immediately either side of it (i.e., Ala347 or Leu349) were present at a protein–protein interface. A number of binding partners and protein structures were identified (Supplementary Fig. S1). The only structure in which the variant was predicted as severely destabilising to both the underlying structure of ACTB and its protein–protein interaction was in Protein Data Bank (PDB) structure 6nbw,^34^ in which Ser348 makes non-bonded contacts with multiple residues in the enzyme NAA80 (N-alpha-acetyltransferase 80) (Supplementary Figs. S2 and S3). These results suggest that, whilst the p.(Ser348Leu) variant may not destabilise the intrinsic structure of actin in isolation, it may affect interactions with specific binding partners, providing a potential explanation for the variant-specific phenotypes observed.

## Discussion

In this study, we identify the *ACTB* p.(Ser348Leu) *de novo* variant as the putative aetiology of NDM in two unrelated individuals. Review of previously reported cases and a comparison with other pathogenic *ACTB* variants shows that NDM has only been identified in individuals with the p.(Ser348Leu) variant, supporting a variant-specific mechanism.

Co-segregation of variants with disease in multiple unrelated families is required to establish genetic causality in rare monogenic disease, with ClinGen and Genomics England requiring at least three unrelated probands.^35^ With the identification of NDM in three individuals heterozygous for this variant (our two cases plus one confirmed case in the literature^32^), our findings support a role of the *ACTB* p.(Ser348Leu) variant in the aetiology of syndromic NDM, expanding the spectrum of clinical features associated with *ACTB*-related disorders. Autosomal dominant disease-causing *ACTB* variants result in a spectrum of disorders collectively referred to as non-muscle actinopathies, with established genotype-phenotype relationships.^36^ Missense variants predicted to result in a gain of protein function typically result in the rare developmental disorder BWS1, characterised by craniofacial features and intellectual disability,^37^ or dystonia-deafness syndrome, characterised by early-onset deafness and later-onset dystonia.^38^ In contrast, loss-of-function variants cause a pleiotropic developmental disorder distinct to BWS1, with recognisable facial gestalt, increased frequency of internal organ malformations, and intellectual disability.^27^ A further clinically separate disorder arises from missense or frameshift variants in exon 5 or 6, leading to early-onset thrombocytopenia, intellectual disability and dysmorphic facial features.^39^

The association of NDM and hyperglycaemia specifically with the *ACTB* p.(Ser348Leu) variant, but not other *ACTB* variants, suggests a variant-specific effect driving the diabetes phenotype. Variant-specific mechanisms are increasingly recognised in neonatal diabetes. For example, the *de novo CNOT1* p.(Arg535Cys) variant is an established cause of NDM and holoprosencephaly.^5^ Similarly, the homozygous *FICD* p.(Arg371Ser) variant causes a syndrome of NDM with neurodevelopmental delay and cataracts,^17^ whereas a different homozygous variant in this gene result in a neurodegenerative phenotype without diabetes.^40^ Similarly, bi-allelic loss-of-function variants in *TARS2* generally cause Combined Oxidative Phosphorylation deficiency-21 (COXPD-21), which does not involve diabetes; however, the homozygous p.(Arg327Gln) variant has been identified in four individuals, all presenting with neonatal diabetes and neurological features overlapping COXPD-21.^16^ Notably, like *ACTB*, these three genes encode ubiquitous proteins that are essential in multiple cell types. The presence of specific missense variants that disrupt beta-cell development or function, while loss-of-function variants do not cause diabetes, highlights important, specific roles for these genes within the beta-cells.

Individuals with the *ACTB* p.(Ser348Leu) variant exhibit a distinct set of clinical features. In addition to the enrichment of NDM, gastrointestinal atresia or stenosis was more common in this group compared to individuals with other pathogenic missense *ACTB* variants (78% vs 3%). Previous reports have suggested the type of severe gastrointestinal complications observed in these patients may be specific to the p.(Ser348Leu) variant,^32^ with three affected individuals with this variant presenting the rare apple-peel intestinal malformation (Table 1). Neurodevelopmental features appeared less frequent in this group (56% vs 95%); however, this may be influenced by 3/9 individuals with the *ACTB* p.(Ser348Leu) variant having died in infancy.

The very low birthweights and early age at diagnosis of diabetes in individuals with the *ACTB* p.(Ser348Leu) variant, coupled with evidence of exocrine insufficiency in the case reported by Hill et al.^32^ suggests that disrupted pancreatic development may underlie the NDM in these patients. These clinical features with extra-pancreatic complications involving gut development (such as intestinal atresia/stenosis) have been observed in other types of syndromic NDM caused by pancreatic developmental defects, including variants in the pancreatic transcription factors *RFX6*, *GATA6* and *PDX1*.^7^^,^^41^^,^^42^ Whilst some of the features observed in patients with *ACTB*-NDM overlap known syndromic NDM subtypes, the variable phenotype observed among patients with the same *ACTB* p.(Ser348Leu) variant warrants further investigations of a larger group of patients with this variant to define the clinical spectrum of the disease.

The *ACTB* gene encodes beta-actin, a ubiquitously expressed protein with essential roles in both the cytoskeleton and nucleus. Under normal conditions, beta-actin interacts with several other proteins to perform functions ranging from cell migration to gene regulation.^43^ The precise mechanism underlying the diabetes phenotype caused by the p.(Ser348Leu) variant is currently unclear, with beta-actin playing key roles in both beta-cell development and function. The actin cytoskeleton is important in pancreatic development where it acts as a primary mechanical regulator of lineage specification and plays a critical role in early endoderm formation.^44^^,^^45^ Consistent with this, *ACTB* expression in human embryonic stem cells differentiating to mature beta-cells peaks at the definitive endoderm stage (data from De Franco et al.^46^; Supplementary Fig. S4). The actin cytoskeleton also has key functions in mature beta-cells, including actin filament barrier formation to prevent insulin secretion at basal states and providing a track for insulin granules to travel toward the cell membrane in stimulated states.^47^^,^^48^ Our in-silico protein modelling and interaction analysis predicts the *ACTB* p.(Ser348Leu) variant to severely destabilise interaction between actin and NAA80, a N-terminal acetyltransferase (Supplementary Figs. S2 and S3). This enzyme acts as a specific posttranslational modifier of actin, crucial for actin cytoskeleton dynamics. In human embryonic stem cells differentiating to mature beta-cells *NAA80* expression is detected during differentiation, peaking at the pancreatic progenitor stage (data from De Franco et al.^46^; Supplementary Fig. S4). Perturbation of this protein–protein interaction by the *ACTB* p.(Ser348Leu) variant could result in failed reorientation of actin filaments, a mechanism that would affect both development of pancreatic beta-cells (and other endoderm-derived organs) and prevent the release of insulin granules in beta-cells.

Supporting a mechanism affecting early development, Tsujimoto et al. recently modelled the effects of the *ACTB* p.(Ser348Leu) variant in a mammalian epithelial cell line and *Xenopus laevis* embryos.^33^ In both models, beta-actin showed reduced localisation to epithelial cell junctions, attributable to impaired binding with the small actin-binding protein PFN1 (Profilin-1). The authors proposed that this defect impacts epithelial cell migration during development, leading to orofacial cleft formation in BWS1. Given *ACTB*’s status as an important ‘housekeeping’ gene, it is possible cell migration may be disrupted during pancreatic development in a similar manner. Notably, PFN1 is also present in a ternary complex with actin and NAA80 in PDB 6nbw, and although Ser348 is not directly involved in this interaction, there is contact with nearby residues suggesting that disruption of actin-NAA80 binding might also impact on the interaction with PFN1. Further functional studies are required to determine if this is indeed the causative mechanism or rather the *ACTB* p.(Ser348Leu) variant has direct impact on beta-cell function.

Our study had some limitations. Whilst the evidence is consistent with the *ACTB* p.(Ser348Leu) variant being linked to NDM, there is currently a lack of functional evidence to explain the mechanism by which the NDM phenotype arises. Our in-silico analysis highlighted NAA80 as a biologically plausible candidate for a role in mediating pathogenesis of the *ACTB* p.(Ser348Leu) variant, however its identification as such reflects only the currently available structural data for actin and its interactions, and it is possible that future studies may identify other possible candidates. Future studies should investigate the *ACTB* p.(Ser348Leu) variant in human models of beta-cell development and function to explore how this variant results in NDM. Also, since we were reliant on clinical data available in literature reports, we had to exclude some potential NDM cases which could not be confirmed in our enrichment analysis of NDM, for example the case with neonatal hyperglycaemia reported by Sibbin et al.^28^ for whom there was no treatment information, or the case with hyperglycaemia reported by Fakhro et al.^31^ Similarly, it is also possible that other cases may have had hyperglycaemia or diabetes, but it was not mentioned in the literature reports. Additionally, potential ascertainment bias and heterogeneity in the comparison group of patients with *ACTB* variants excluding p.(Ser348Leu) may limit the conclusions we can draw from our enrichment analysis. Identification of additional individuals with *ACTB*-NDM will be essential to further delineate the phenotype and genetic features of this novel NDM subtype.

Overall, our study highlights a putative role of the *ACTB* p.(Ser348Leu) variant in the aetiology of syndromic NDM, likely through a variant-specific mechanism. As a result, we recommend the *ACTB* gene is added to routine genetic testing for NDM.

## Contributors

E.D.F. conceived and designed the study. M.T., K.P., A.T.H., P.B., S.E.F., and E.D.F. recruited the patients and analysed clinical data. M.N.W. performed initial processing of genome sequencing data. S.A., V.L., J.R.S., and E.D.F. analysed and interpreted genome sequencing and targeted next generation sequencing data. R.C. performed in silico protein modelling. M.T. and M.V. provided clinical information. S.A. and V.L. curated the clinical information. S.A. created display items and wrote the first manuscript draft, which was critically revised by E.D.F., S.E.F., and P.B. with input from all authors. E.D.F. and S.A. had full access to all the data in the study and take responsibility for the integrity of the data and the accuracy of the data analysis. All authors read and approved the final version of the manuscript.

## Data sharing statement

Clinical and genotype data can be used to identify individuals and is therefore available through collaboration to experienced teams working on approved studies examining the mechanisms, cause, diagnosis and treatment of diabetes and other beta-cell disorders. Requests for collaboration will be considered by a steering committee following an application to the Genetic Beta-cell Research Bank (https://www.diabetesgenes.org/current-research/genetic-beta-cell-research-bank/). Contact by email should be directed to Prof Elisa De Franco (e.de-franco@exeter.ac.uk). All requests for access to data will be responded to within 14 days.

## Declaration of interests

The authors declare no conflict of interest.
