# Supplementary material for: Identification of the ACTB p.Ser348Leu de novo variant in individuals with syndromic neonatal diabetes
Source: eBioMedicine. 2026 May 9;128:106286. doi: 10.1016/j.ebiom.2026.106286 (PMC13191097; doi:10.1016/j.ebiom.2026.106286)

**Supplementary material**

Table of Contents

**Supplementary table S1. Missense variants and their associated phenotypes in Figure 12**

**Supplementary figure S1. Analysis of protein-protein interactions involving ATCB Ser3483**

**Supplementary figure S2. Structure of ACTB in complex with N-α-acetyltransferase 80 and Profilin-14**

**Supplementary figure S3. Predicted impact of the ACTB Ser348Leu variant5**

**Supplementary Figure S4. Expression of ACTB (left) and NAA80 (right) across development of embryonic stem cell-derived pancreatic islets6**

**Supplementary table S1.**
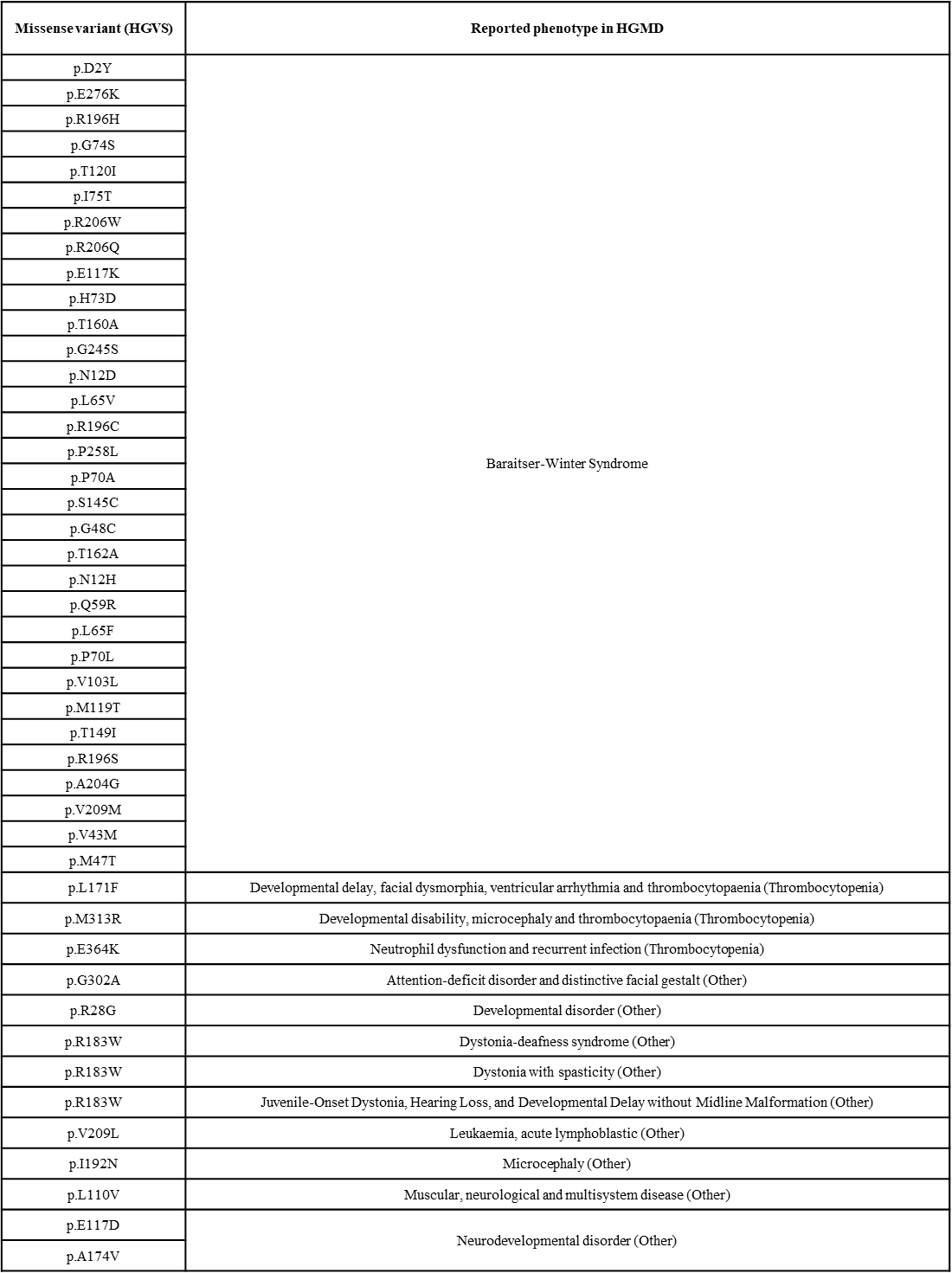
 **Missense variants and their associated phenotypes in Figure 1.** Missense variants are listed in HGVS formats alongside their reported in HGMD. Phenotypes classified as ‘thrombocytopenia’ or ‘other’ in Figure 1 are indicated in brackets. The *ACTB* p.S348L variant is excluded from the table. HGVS= Human genome variation society, HGMD= Human gene mutation database.

**
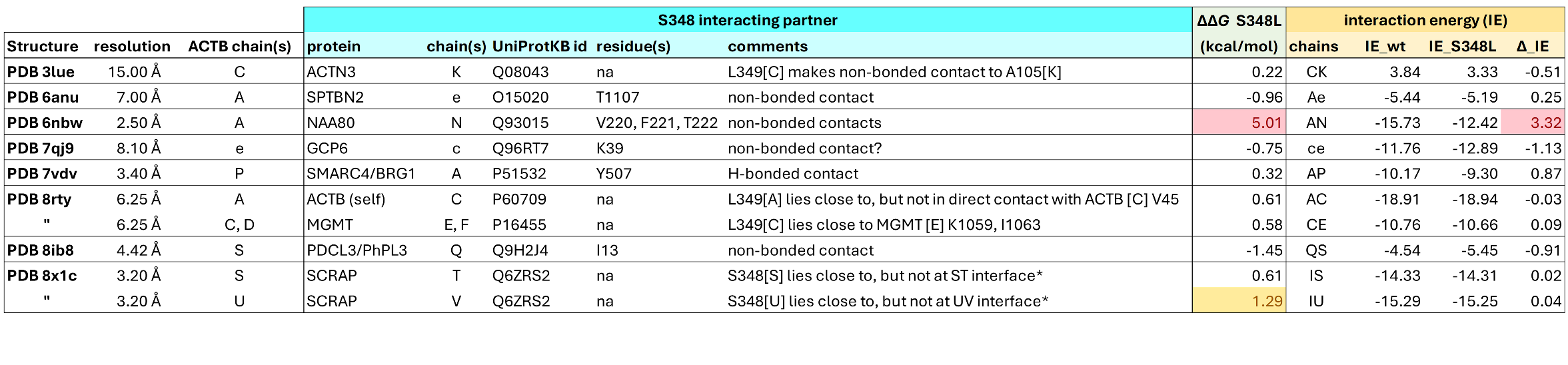
**

**Supplementary figure S1: Analysis of protein-protein interactions involving ATCB Ser348.** The PDBe-KB database was inspected to identify Protein Data Bank (PDB) entries in which Ser348 and/or its neighbours Ala347 or Leu349 are involved in protein-protein interactions. PDB entries identified are listed, along with details of the structure and reported interactions of Ser348 or Leu349 (Ala347 is buried and not directly involved in any protein-protein interactions). The p.(Ser348Leu) variant was then introduced into ACTB chains in all structures by *in silico* mutagenesis using FoldX v5.0, yielding values for ΔΔ*G*, the change in free energy of the variant structure compared to that of the native PDB structure, in kcal/mol; values are reported for the overall change in free energy of the structure due to the variant (ΔΔ*G* S348L), the interaction energy between either wild type ACTB or the p.(Ser348Leu) variant and its binding partner (columns IE_wt or IE_S348L, respectively), and the change in interaction energy due to the variant (column Δ_IE). Values of ΔΔ*G* and Δ_IE were classified according to widely used thresholds as follows: <1 kcal/mol, neutral or benign (no shading); 1-3 kcal/mol, destabilising (yellow shading); >3 kcal/mol, severely destabilising (red shading); a severely destabilising effect of the variant was observed only in PDB 6nbw for the interaction with NAA80. * ST Interface represents the interaction between Chain S (ACTB) and Chain T (SCRAP); UV Interface represents the interaction between Chain U (also ACTB) and Chain V (also SCRAP); both interfaces are structurally equivalent in PDB structure 8x1c.


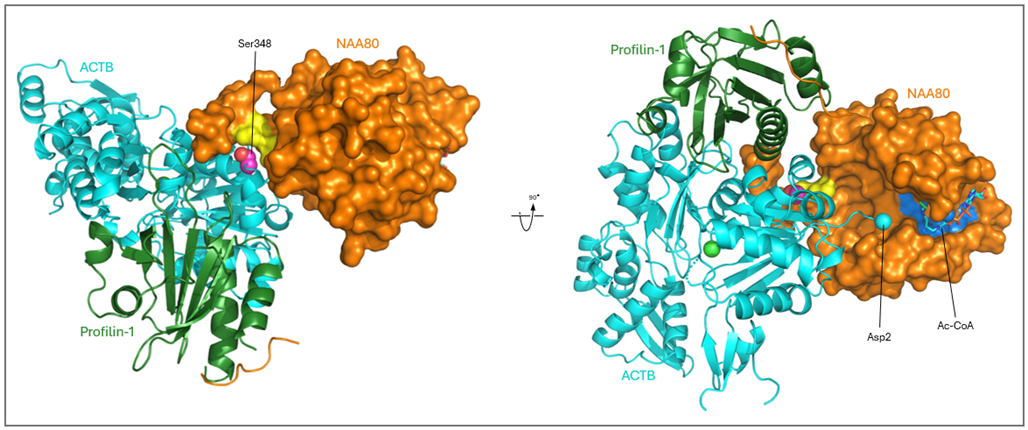


**Supplementary figure S2. Structure of ACTB in complex with N-α-acetyltransferase 80 and Profilin-1.** Figures show PDB 6nbw, the structure of ACTB (cyan ribbon) in complex with N-α-acetyltransferase 80 (NAA80; orange surface) and Profilin-1 (green ribbon); sidechain atoms of Ser348 are shown as space-filling spheres, with carbon atoms coloured magenta; the α-carbon of ACTB Asp2 is also shown as a sphere, bound in the active site cleft of NAA80, while the green sphere shows a calcium ion bound by ACTB; in NAA80, residues Val220 and Thr222, which make non-bonded contact with ACTB Ser348, are coloured yellow, while residues of the acetyl-CoA (Ac-CoA) binding pocket are coloured blue; the Ac-CoA molecule is superimposed from PDB 6nas, which shows NAA80 in complex with rabbit ACTB; in Ac-CoA, carbon atoms of the acetyl group (proximal to ACTB Asp2) are coloured green, with those of the CoA group in cyan.


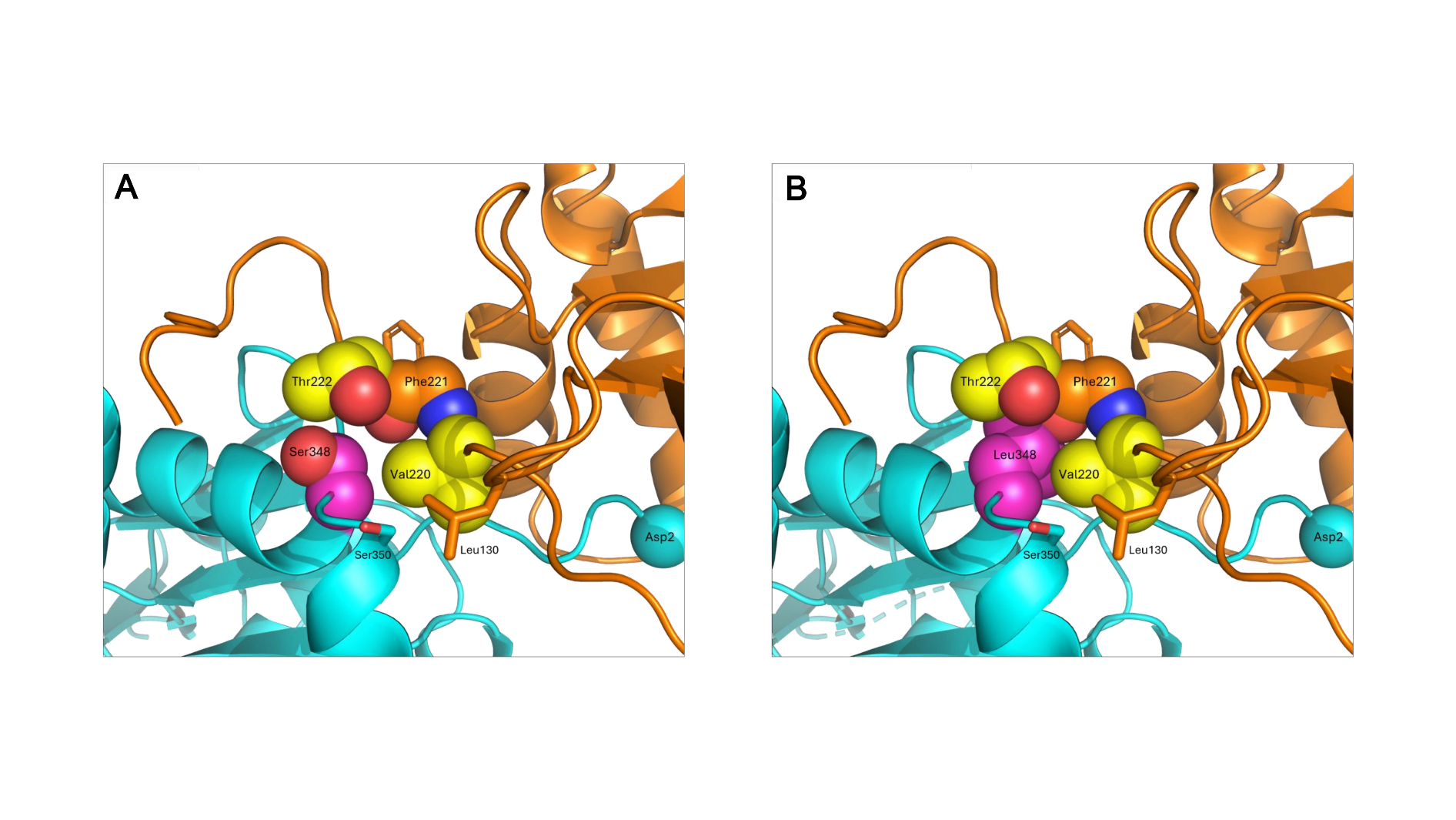


**Supplementary figure S3. Predicted impact of the ACTB Ser348Leu variant.** **(A)** PDB 6nbw, as shown in the previous slide but focussed on Ser348 and the interface with NAA80; in NAA80, sidechain atoms of Val220 and Thr222 are shown as spheres, with carbon atoms coloured yellow; spheres are also shown for backbone atoms of Phe221 (orange), with the sidechain shown in stick format. **(B)** As A, but following introduction of the Ser348Leu variant by in silico mutagenesis; the novel leucine sidechain is predicted to make steric clashes with sidechain atoms of Val220 and Thr222, and with backbone atoms of Phe221; ΔΔ*G*, the change in free energy of the variant structure compared with that of the native, was calculated as 5.01 kcal/mol, with a reduction in the interaction energy between ACTB and NAA80 of 3.32 kcal/mol.

**Supplementary Figure S4. Expression of *ACTB* (left) and *NAA80* (right) across development of embryonic stem cell-derived pancreatic islets.** Expression data comes from RNA sequencing performed on wild type cells in *De Franco et al*^44^*.*


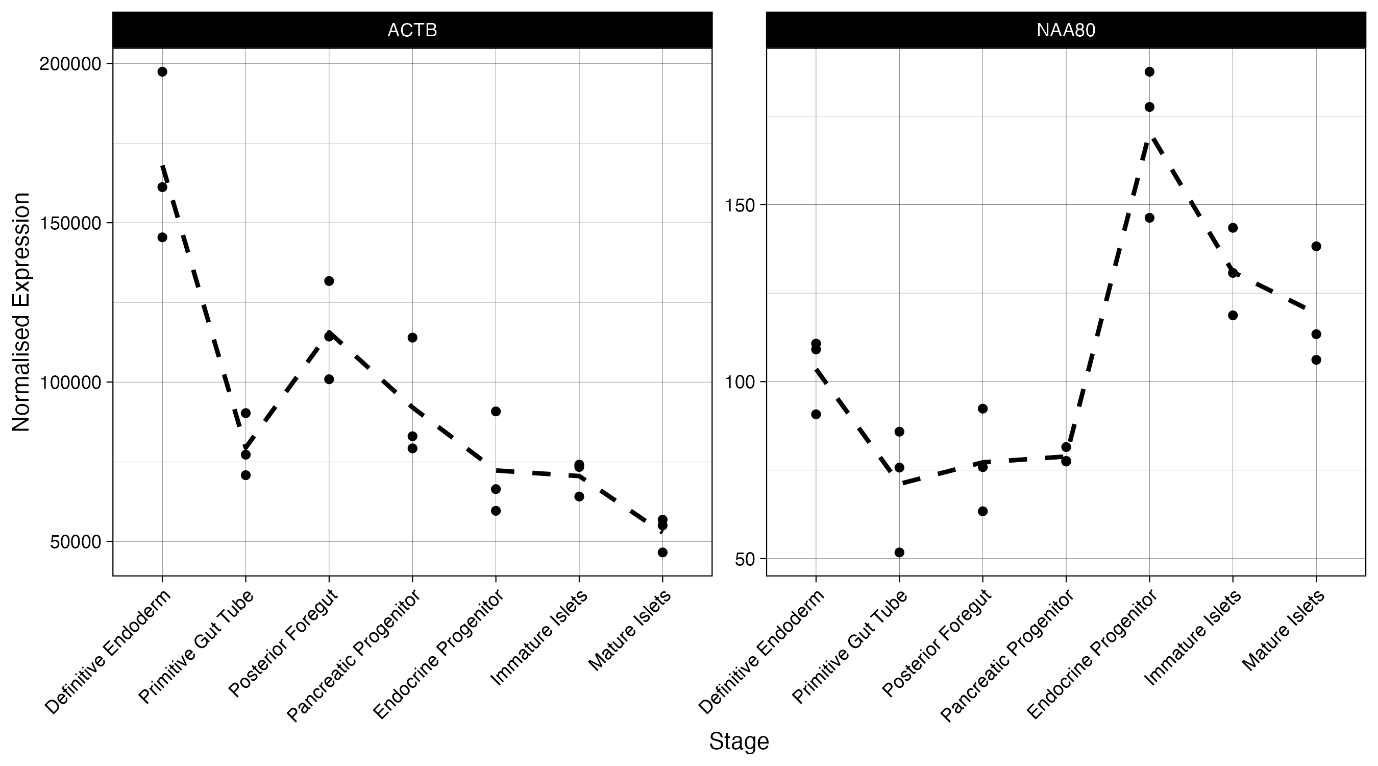

Supplement: Supplementary Figs. S1–S4 and Table S1 [file mmc1.docx]
